# Incidence and Factors Associated With Second Primary Invasive Melanoma in Norway

**DOI:** 10.1001/jamadermatol.2023.6251

**Published:** 2024-02-28

**Authors:** Reza Ghiasvand, Adele C. Green, Marit B. Veierød, Trude E. Robsahm

**Affiliations:** 1 Department of Research, Cancer Registry of Norway, Oslo, Norway; 2 Oslo Centre for Biostatistics and Epidemiology, Oslo University Hospital, Oslo, Norway; 3 Institute for Cancer Research, Oslo University Hospital, Oslo, Norway; 4 Department of Population Health, QIMR Berghofer Medical Research Institute, Brisbane, Queensland, Australia; 5 Cancer Research UK Manchester Institute, University of Manchester, Manchester, United Kingdom; 6 Oslo Centre for Biostatistics and Epidemiology, Institute of Basic Medical Sciences, Department of Biostatistics, University of Oslo, Oslo, Norway

## Abstract

**Importance:**

Patients diagnosed with a primary melanoma are at high risk of subsequent melanomas. Understanding the risk of second primary invasive melanoma and associated factors is crucial to optimize patient follow-up.

**Objective:**

To assess the incidence rate of second primary invasive melanoma and time between the first and second primary invasive melanoma in the Norwegian population.

**Design, Setting, and Participants:**

This population-based cohort study included data from deidentified records of all invasive melanomas diagnosed in Norway in 2008 to 2020, obtained from the Cancer Registry of Norway. Data were from adults aged 18 years or older diagnosed with a first primary melanoma. Data analysis was performed from March to August 2023.

**Main Outcomes and Measures:**

The main outcome was the incidence rate of second primary invasive melanoma at least 30 days after the first. Accelerated failure time models were fitted to examine potential associations with patient and tumor characteristics. Median time between first and second primary melanomas and 95% CIs were calculated. The likelihood of, and median interval for, second primary melanomas on the same or different site as the first primary were calculated.

**Results:**

A total of 19 196 individuals aged 18 years or older were diagnosed with a first primary melanoma. The mean (SD) age at diagnosis of the first primary melanoma was 62 (16) years (range, 18-104 years), and 9763 (51%) were female. The incidence rate in the year following diagnosis was 16.8 (95% CI, 14.9-18.7) per 1000 person-years, which decreased to 7.3 (95% CI, 6.0-8.6) during the second year and stabilized thereafter. Median time between first and second primaries decreased with advancing age and was 37 months (95% CI, 8-49) in patients younger than 40 years, 18 (95% CI, 13-24) in patients aged 50 to 59 years, and 11 (95% CI, 7-18) in patients aged 80 years or older. The second primary was on the same site as their first primary for 47% (359 patients), and on a different site for 53% (407 patients). The median interval until second melanoma on the same site as the initial melanoma was 12 (95% CI, 7-19) months in men and 22 (95% CI, 11-35) months in women.

**Conclusions and Relevance:**

Older age and male sex were associated with an increased risk, suggesting that increased surveillance intensity may be considered for men, especially those older than 50 years, for at least 3 years after their initial diagnosis, regardless of the characteristics of their first invasive melanoma.

## Key Points

**Question:**

What is the incidence rate of second primary invasive melanoma and when is it most likely to occur?

**Findings:**

In this cohort study of 19 196 individuals aged 18 years or older diagnosed with a first primary melanoma, the incidence rate of second primary melanoma was highest in the year following diagnosis of the first primary melanoma, decreasing in the second year, and stabilizing thereafter. Older age (aged ≥50 years) and male sex were associated with increased risk and shorter interval before second primary melanoma occurrence.

**Meaning:**

The findings of this study suggest that increased surveillance intensity may be considered in the first 3 years, especially in older men.

## Teaser

This cohort study assesses the incidence rate of second primary invasive melanoma at least 30 days after the first based on data from deidentified records of all invasive melanomas diagnosed in 2008 to 2020, obtained from the Cancer Registry of Norway.

## Introduction

The steep increase in the incidence of cutaneous melanoma, along with larger numbers of melanomas being diagnosed at earlier stages, and improvements in melanoma treatment have resulted in an expanding population of melanoma survivors over the past decade.^1,2^ These patients are at risk of developing subsequent melanomas during their lifetime,^3^ although the reported risk of second primary melanoma from different studies varies substantially.^4,5,6,7,8,9,10,11,12,13^ Some previous studies^4,5,6,7,8,9,10,11,12,13^were limited to a single center, some combined in situ and invasive melanomas, and others included both synchronous (ie, second primary melanoma within the 30 days of their first diagnosis) and metachronous (ie, second primary melanoma after 30-day interval of their first diagnosis) primary melanomas. It is crucial to differentiate between the 2 types of lesions when studying risk of subsequent melanoma, as it is metachronous lesions that are of concern during patient follow-up in clinical practice.^4,10^

Regular follow-up of patients with melanoma allows early detection of any signs of disease progression and any new melanomas. In contrast, prolongation of the follow-up period may result in overdiagnosis, overtreatment, anxiety in patients, and increased clinical workload and associated costs. Thus, understanding how the risk for subsequent primary melanomas changes over time would enable clinicians to optimize follow-up time and tailor surveillance and management strategies for melanoma survivors. However, knowledge of changes in the risk of second primary over time after the first diagnosis and the optimal follow-up time is incomplete.

Previous studies have reported an increased risk of second primary melanoma associated with older age, male sex, family history of melanoma, a high number of nevi, recreational sun exposure, and higher ambient UV radiation at the place of residence.^3,9,10,11,14^ However, it remains unclear whether second primary melanoma tends to occur on the same body site as the first primary. To our knowledge, neither the likelihood and possible time differences between the development of a second primary on the same body site compared with a different body site have been reported.

Using nationwide data from the Cancer Registry of Norway (CRN), we aimed to estimate the incidence rate and time between the initial diagnosis and second primary melanoma, to explore the role of patient and tumor characteristics, and to estimate the likelihood and possible heterogeneity of time interval between second primaries on the same body site as the first primary vs those that occurred on different body sites.

## Methods

### Patients

We obtained deidentified records of all invasive melanomas, *International Statistical Classification of Diseases and Related Health Problems, Tenth Revision (ICD-10)* code C43, diagnosed in Norway from 2008 to 2020 from the CRN. Mandatory reporting of all cancer diagnoses, except basal cell carcinoma, ensures complete and high-quality data in the CRN, whereby more than 99% of melanomas diagnosed after 2000 are morphologically verified.^15^ In 2008 the CRN established the Norwegian Melanoma Registry, which provided additional data on tumor thickness, ulceration, and TNM staging. While information about the ethnic background of patients was not available, the overall population of Norway is White (93%).^16^ Permission to conduct the study and ethical approval were obtained from the Norwegian Data Inspectorate at the national level, the Southeast Norway Regional Committee for Medical and Health Research Ethics, and the relevant registries. Data analysis was performed from March to August 2023. This study followed the Strengthening the Reporting of Observational Studies in Epidemiology (STROBE) reporting guideline.

### Outcome and Follow-Up

The main study outcome was the diagnosis of a second primary invasive melanoma at least 30 days after the first primary melanoma. Patients diagnosed with synchronous melanomas were not considered to have a second primary melanoma for the purposes of this study. Follow-up time was calculated from the date of the first primary melanoma diagnosis to 10 years after this date, the date of the second primary melanoma diagnosis, death, or end of the study period (December 2020), whichever occurred first.

### Variables for Analysis

Tumor site was categorized as head and neck (*International Classification of Diseases for Oncology, Third Revision* [*ICD-O-3*] codes C43.0-C43.4), trunk (C43.5), upper limb (C43.6), lower limb (C43.7), and unspecified (C43.9). The agreement in anatomical site between the first and second primary melanomas was determined by whether they occurred on the same site, the same site but on the opposite side, or a different site. While we had laterality data for all tumor sites, we lacked information regarding whether tumor sites on limbs were distal or proximal. Morphologic subtypes were categorized according to *ICD-O-3* codes, as superficial spreading melanoma (SSM: 87433), nodular melanoma (NM: 87213), lentigo maligna melanoma (LMM: 87423), other subtypes (87223, 87233, 87303, 87403, 87443, 87453, 87613, 87703, 87713, 87723, 87803), and unspecified (80003, 872003). Tumor thickness was categorized according to the American Joint Committee on Cancer Eighth Edition (AJCC 8) staging as T1: ≤1.0 mm, T2: >1.0-2.0 mm, T3: >2.0-4.0 mm, and T4: >4.0 mm.^17^ Ulceration was categorized as absent, present, or unspecified. Metastasis was categorized as absent, present, or unspecified. The TNM stage was defined according to AJCC 8.^17^ In Norway, there is a noticeable gradient in ambient UV radiation (UVR) with decreasing levels from southern to northern latitudes. Therefore, information on the region of residence was used as a proxy for ambient UVR exposure and divided into 3 categories: highest (eastern and southern Norway), medium (western and central Norway), and lowest (northern Norway) levels.

### Statistical Analysis

The incidence rate and 95% CIs of second primary invasive melanoma during the 10-year period following the first primary diagnosis were calculated per 1000 person-years. The probability (calculated as the mean probability of second primary melanoma during the follow-up period)^18^ by different follow-up time points and different factors was calculated. Additionally, we fitted accelerated failure time models to examine potential patient and tumor characteristics associated with the development of a second primary melanoma. Multivariable models were adjusted for all patient and tumor characteristics as well as the calendar year at diagnosis of first primary melanoma. Rate ratios (RRs) and 95% CIs have been reported. We tested whether the association between patient and tumor characteristics and risk of second primary melanoma differed by sex, using the likelihood ratio test. For patients who developed a second primary melanoma, median time intervals (ie, the time point at which 50% of patients had developed a second primary) with corresponding 95% CIs were calculated, overall and by sex and tumor site. These estimates were also calculated separately for patients who developed the second primary at the same location as their first primary, those who developed it on the contralateral site, and those who had it on another site. *P* values were 2-sided and considered significant at *P* < .05. All statistical analyses were performed using Stata, version 17 (StataCorp LLC).

## Results

A total of 19 329 individuals aged 18 years or older were diagnosed with a first primary melanoma in Norway between January 2008 and December 2020. We excluded 75 patients who were lost to follow-up and 57 patients who died in the same month they were diagnosed. The final study sample consisted of 19 196 patients, of whom 9763 (51%) were female and 9433 (49%) were male. The mean (SD) age at diagnosis of the first primary melanoma was 62 (16) years (range, 18-104 years). During 98 000 person-years of follow-up (median 4.6 years; range, <1-10), 766 (4%) patients, including 326 (43%) women and 440 (57%) men, developed a second primary melanoma. The mean (SD) age at diagnosis of the second primary was 67 (13) years (range, 20-98 years).

### Incidence of Second Primary Melanoma

The probability of developing a second primary melanoma in the year following the first primary was 16.5 (95% CI, 14.8-18.5) per 1000 patients (ie, on average 16.5 of 1000 patients would develop a second primary within the first year) (Table 1). The mean probability was 8.1 (95% CI, 7.5-8.8) per 1000 patients per year during the first 5 years and 6.4 (95% CI, 5.9-6.9) per 1000 patients per year within 10 years of follow-up.

**Table 1.** Probability of Second Primary Melanoma and Rate Ratios (RRs) for Patient and Tumor Characteristics

Variable	Patients, No. (%)^a^	Events, No. (%)	Probability (95% CI)^b^	RR (95% CI)^c^	Adjusted RR (95% CI)^d^
Follow-up, y					
1	19 196 (100)	312 (41)	16.5 (14.8-18.5)	NA	NA
2	19 196 (100)	434 (57)	11.9 (10.8-13.1)	NA	NA
5	19 196 (100)	634 (83)	8.1 (7.5-8.8)	NA	NA
10	19 196 (100)	766 (100)	6.4 (5.9-6.9)	NA	NA
Sex					
Female	9763 (51)	326 (43)	5.1 (4.5-5.8)	1 [Reference]	NA
Male	9433 (49)	440 (57)	7.8 (7.0-8.8)	1.52 (1.32-1.76)	1.37 (1.18-1.59)
Age at diagnosis, y					
<40	1762 (9)	7 (5)	3.0 (2.2-4.3)	1 [Reference]	1 [Reference]
40-49	2771 (14)	76 (10)	4.0 (3.1-5.2)	1.37 (0.93-2.03)	1.32 (0.89-1.96)
50-59	3714 (19)	130 (17)	5.5 (4.5-6.7)	1.86 (1.29-2.68)	1.73 (1.20-2.49)
60-69	4593 (24)	228 (30)	7.8 (6.8-9.1)	2.69 (1.90-3.81)	2.49 (1.76-3.53)
70-79	3786 (20)	186 (24)	8.0 (6.7-9.1)	3.16 (2.22-4.49)	2.82 (1.98-4.02)
≥80	2570 (13)	109 (14)	9.6 (7.3-12.6)	3.59 (2.47-5.21)	3.29 (2.25-4.82)
Place of residence					
Southern Norway	15 541 (81)	610 (80)	6.3 (5.8-7.0)	1 [Reference]	1 [Reference]
Central Norway	2463 (13)	109 (14)	6.7 (5.4-8.4)	1.15 (0.94-1.42)	1.20 (0.96-1.48)
Northern Norway	1174 (6)	47 (6)	6.1 (4.3-8.4)	1.03 (0.76-1.39)	1.07 (0.78-1.44)
Missing	18	0	NA	NA	NA
Body site					
Trunk	7693 (40)	354 (46)	7.3 (6.4-8.2)	1 [Reference]	1 [Reference]
Lower limb	4569 (24)	165 (22)	5.5 (4.6-6.5)	0.76 (0.63-0.92)	0.87 (0.73-1.07)
Upper limb	3742 (20)	142 (19)	6.0 (4.9-7.2)	0.84 (0.69-1.02)	0.82 (0.67-1.01)
Head and neck	2334 (12)	95 (12)	6.8 (5.4-8.7)	0.99 (0.79-1.25)	0.86 (0.68-1.10)
Unspecified	858 (4)	10 (1)	2.9 (1.4-5.7)	0.40 (0.22-0.76)	0.42 (0.19-0.91)
Subtype					
Superficial spreading	10 851 (57)	455 (59)	6.5 (5.8-7.2)	1 [Reference]	1 [Reference]
Nodular	3214 (17)	129 (17)	6.5 (5.3-7.9)	1.09 (0.90-1.33)	0.84 (0.67-1.07)
Lentigo maligna	606 (3)	26 (3)	7.9 (4.9-12.8)	1.17 (0.79-1.74)	0.94 (0.62-1.42)
Other subtypes	4485 (23)	154 (20)	5.8 (4.8-7.0)	0.90 (0.75-1.08)	0.88 (0.72-1.07)
Unspecified	40	2	5.3 (1.3-2.1)	1.72 (0.41-7.18)	1.43 (0.33-6.28)
Tumor thickness^e^					
T1 (≤1.0 mm)	9852 (51)	365 (48)	5.7 (5.0-6.4)	1 [Reference]	1 [Reference]
T2 (>1.0-2.0 mm)	3863 (20)	201 (26)	7.8 (6.6-9.1)	1.46 (1.23-1.74)	1.43 (1.19-1.72)
T3 (>2.0-4.0 mm)	2314 (12)	93 (12)	6.4 (4.9-8.2)	1.25 (1.00-1.58)	1.18 (0.74-1.87)
T4 (>4.0 mm)	1755 (9)	70 (9)	7.7 (5.6-10.5)	1.69 (1.31-2.19)	1.46 (0.90-2.36)
Missing	1412 (7)	37 (5)	6.4 (4.3-9.4)	1.02 (0.73-1.43)	1.49 (0.78-2.84)
Ulceration					
Absent	12 671 (66)	479 (63)	6.6 (5.8-7.4)	1 [Reference]	1 [Reference]
Present	2736 (14)	114 (15)	7.9 (6.3-9.9)	1.26 (1.02-1.55)	1.02 (0.77-1.33)
Unspecified	3789 (20)	173 (22)	5.7 (4.9-6.7)	0.81 (0.78-0.96)	0.87 (0.71-1.07)
Metastasis					
Absent	16 067 (84)	676 (88)	6.6 (6.0-7.2)	1 [Reference]	1 [Reference]
Regional metastasis	1182 (6)	46 (6)	8.3 (5.1-13.4)	1.44 (1.07-1.95)	1.01 (0.61-1.68)
Distant metastasis	931 (5)	6 (1)	1.3 (0.5-2.9)	0.32 (0.14-0.73)	0.24 (0.08-0.75)
Unspecified	1016 (5)	38 (5)	4.8 (3.5-6.7)	0.67 (0.48-0.93)	0.76 (0.53-1.07)
Stage^f^					
I	12 958 (67)	531 (69)	6.1 (5.8-6.8)	1 [Reference]	1 [Reference]
II	4328 (23)	188 (25)	7.3 (6.1-8.7)	1.27 (1.07-1.50)	0.98 (0.62-1.53)
III	723 (4)	27 (4)	6.7 (3.9-11.3)	1.65 (1.11-2.44)	1.18 (0.74-1.89)
IV	1183 (6)	20 (3)	5.3 (2.9-9.6)	0.73 (0.47-1.15)	0.71 (0.33-1.53)
Missing	4	0	NA	NA	NA

Abbreviation: NA, not applicable.

^a^ Number of patients at the start of follow-up. Percentage not reported for values less than 1%.

^b^ Average 10-year probability of developing second primary melanoma per 1000 patients per year, except when the follow-up period is specified.

^c^ Parametric accelerated failure time models with exponential distribution.

^d^ Parametric accelerated failure time models with exponential distribution, adjusted for all characteristics in the table as well as the calendar year of first primary melanoma diagnosis.

^e^ Tumor thickness was categorized according to American Joint Committee on Cancer Eighth Edition (AJCC 8) staging.

^f^ The TNM stage was defined according to AJCC 8.

**Figure 1.**
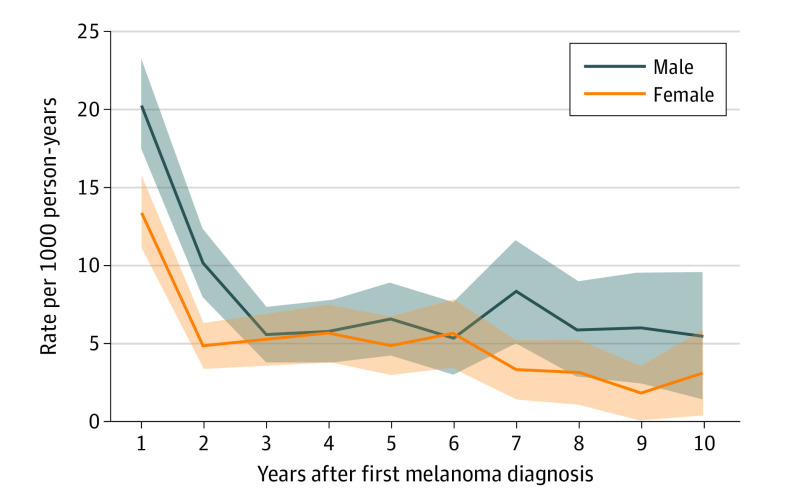
Second Primary Melanoma per 1000 Person-Years in Patients Diagnosed With a First Primary Melanoma in Norway Between 2008 and 2020 The shaded areas indicate 95% CIs.

The incidence rate of a second primary during the first year of follow-up was 16.8 (95% CI, 14.9-18.7) per 1000 person-years, which decreased to 7.3 (95% CI, 6.0-8.6) per 1000 person-years during the second year of follow-up and remained relatively stable during and after the third year from the diagnosis of the first primary melanoma (eTable 1 in Supplement 1). The incidence rate during the first year of follow-up was higher for men (20.3; 95% CI, 17.4-23.3) per 1000 person-years than for women (13.5; 95% CI, 11.1-15.8) per 1000 person-years, and this difference continued throughout the follow-up period (Figure 1; eTable 1 in Supplement 1).

### Risk Factors for a Second Primary Melanoma

**Figure 2.**
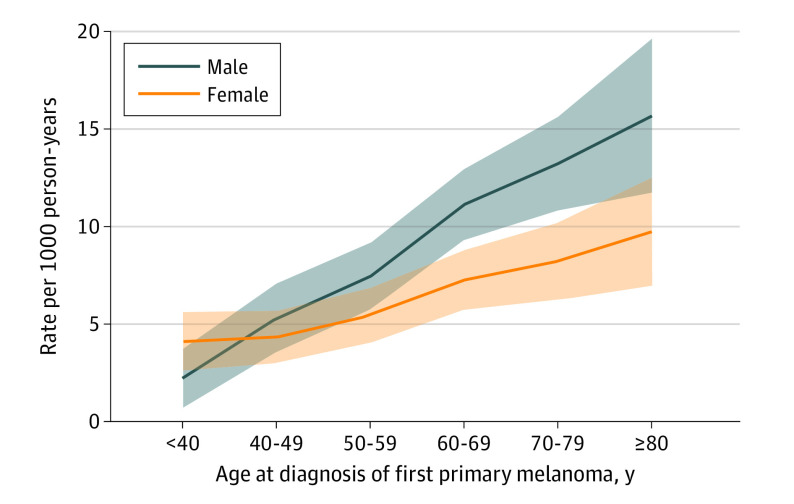
Second Primary Melanoma per 1000 Person-Years by Sex and Age at Diagnosis in Patients Diagnosed With a First Primary Melanoma in Norway Between 2008 and 2020 The shaded areas indicate 95% CIs.

Male sex was associated with an increased risk of second primary melanoma (adjusted RR, 1.37; 95% CI, 1.18-1.59) (Table 1). Older age was also associated with increased risk, and in multivariable analysis, patients aged 50 years or older had a significantly higher risk of developing a second primary melanoma compared with patients aged younger than 40 years. Moreover, we found interaction between age and sex on risk of second primary melanoma, and while the incidence rate of a second primary was 2 times higher for women aged 80 years or older compared with women aged younger than 40 years (adjusted RR, 2.11; 95% CI, 1.31-3.42), the rate was more than 7 times higher for their male counterparts (adjusted RR, 7.33; 95% CI, 3.46-15.51) (Figure 2; eTable 2 in Supplement 1). We found no association between place of residence and risk of second primary melanoma.

In univariable analysis, melanoma located on lower limbs was associated with a decreased risk of a second primary compared with trunk melanoma (Table 1). Also, first primaries greater than or equal to T2 vs T1, ulcerated vs unnucleated, first primaries with regional metastasis compared with no metastasis, and TNM stages II and III vs I were associated with increased risk, while distant metastasis was associated with a decreased risk of second primary melanoma (Table 1). However, in the multivariable analysis, only tumor thickness T2 vs T1 was associated with an increased risk of second primary melanoma (adjusted RR, 1.43; 95% CI, 1.19-1.72) and distant metastasis was associated with a decreased risk (adjusted RR, 0.24; 95% CI, 0.08-0.75) (Table 1).

### The Median Time Between a First and Second Primary Melanoma

Among those affected, the median interval before the second primary melanoma (ie, the time in which 50% of patients developed a second primary melanoma) was 17 months (95% CI, 15-20 months) (Table 2). The median time was 20 months (95% CI, 15-27 months) for women and 17 months (95% CI, 14-19 months) for men. The median interval decreased with an increase in age, and while the median interval for the age group younger than 40 years was 37 months (95% CI, 8-49 months), it was 18 months (95% CI, 13-24 months) for patients aged 50 to 59, and 14 months (95% CI, 9-19 months) for patients aged 70 to 79 years and 11 (95% CI, 7-18) in patients aged 80 years or older.

**Table 2.** Median Interval Between a First and Second Primary Melanoma and Median Thickness of Second Melanoma

Variable	No. (%)	Median interval, mo (95% CI)	Median thickness, mm (range)
Overall	766 (100)	17 (15-20)	0.7 (0.5-1.2)
Sex			
Female	326 (43)	20 (15-27)	0.7 (0.4-1.2)
Male	440 (57)	17 (14-19)	0.8 (0.5-1.3)
Age, y			
<40	37 (5)	37 (8-49)	0.7 (0.4-1.0)
40-49	76 (10)	24 (16-34)	0.6 (0.4-1.0)
50-59	130 (17)	18 (13-24)	0.7 (0.5-1.1)
60-69	228 (30)	21 (15-28)	0.8 (0.5-1.2)
70-79	186 (24)	14 (9-19)	0.8 (0.4-1.1)
≥80	109 (14)	11 (7-18)	1.2 (0.6-3.3)
Body site			
Same site	169 (22)	16 (11-21)	0.8 (0.5-1.2)
Female	76 (10)	22 (11-35)	0.7 (0.4-1.0)
Male	93 (12)	12 (7-19)	0.9 (0.5-1.2)
Same site, opposite side	190 (25)	21 (14-32)	0.7 (0.5-1.2)
Female	68 (9)	23 (11-36)	0.6 (0.5-1.2)
Male	122 (16)	19 (12-31)	0.7 (0.5-1.2)
Different site	407 (53)	18 (15-21)	0.8 (0.4-1.4)
Female	182 (24)	17 (13-27)	0.7 (0.4-1.2)
Male	225 (29)	18 (14-20)	0.8 (0.5-1.5)

### Second Primary at the Same Body Site vs Different Site

In patients who experienced a second primary melanoma, 47% (359 patients) developed it on either the same (22%) or the opposite side (25%) of the same site as their first primary, while 53% (407 patients) had it on a different body site (Table 2). The median interval was shorter for men who developed a second primary on the same site (median interval, 12 months; 95% CI, 7-19 months) than in women (median interval, 22 months; 95% CI, 11-35 months). Median interval was comparable for second primaries developing on the contralateral side (21 months; 95% CI, 14-32 months), and those developing on a different site (18 months; 95% CI, 15-21 months), with a similar pattern of median interval between men and women. Moreover, the median tumor thickness of the second primary melanoma was comparable between second tumors on the same, contralateral, and different sites (Table 2).

## Discussion

In this nationwide cohort study, 4% of patients with a first primary melanoma developed a second primary. Our findings suggest that 17 of 1000 patients with melanoma will develop a second primary melanoma within the first year, and on average, 6.4 of 1000 patients will develop a second primary invasive melanoma every year within the first 10 years. The incidence rate of second primary melanomas was highest in the first year of follow-up, decreased in the second year, and stabilized thereafter. The incidence rates were higher for men than for women. Age greater than 50 years and male sex were associated with an increased risk. Moreover, the incidence rate increase associated with older age was more prominent for men than women. We did not find any association between our geographic proxy for ambient UVR exposure or tumor characteristics of initial melanoma and risk of a second primary melanoma. Among patients who developed a second primary melanoma, the median time interval between first and second primary melanoma was considerably shorter for older patients than younger patients. The median interval was shorter in men who developed a second primary on the same site as their first primary, but not in women. Median tumor thickness of the second primary was comparable between patients with a second primary on the same site and on a different site.

The proportion of patients developing a second primary melanoma ranges from 2% to 20% in previous studies.^19,20,21,22,23,24,25^ This variation may be explained by different study designs, different inclusion criteria, and varying lengths of follow-up. Moreover, many of these studies combined in situ and invasive melanomas,^26^ which made their results more vulnerable to bias due to overdiagnosis, increased patient awareness, and increased screening scrutiny in affected populations.^12^ In contrast, we included only invasive second primary melanomas in our study to minimize the impact of overdiagnosis.

Male sex and advancing age were independent factors associated with second primary melanoma in our study, which aligns with previous reports.^13,21^ Additionally, we observed that sex and age also interact with each other and higher incidence rate of second primary in older patients was more prominent in men than women.

An association between ambient UVR and occurrence of first primary melanoma in Norway has been documented previously.^27,28^ However, we found no association between latitude of residence and risk of a second primary melanoma. One explanation might be that those who develop a melanoma are more likely to have comparable personal UVR exposure and genetic predisposition, and thus similar risk of a second primary regardless of their ambient UVR.

Similar to our results, several previous studies found no association between the tumor characteristics of the initial melanoma and risk of second primary melanoma.^10,13,21^ However, a higher risk of second primary melanoma associated with the first melanoma diagnosis on the head or neck or the trunk has been reported.^10^ In the present study, patients with their first primaries on the trunk or head or neck had a higher probability of second primary; however, after adjusting for sex and age in multivariable analysis, the association disappeared. The negative association between distant metastasis and risk of a second primary melanoma in our study reflects poor survival in these patients after their first primary melanoma.

A few previous studies compared the concordance in tumor location between first and second primary melanomas and reported modest correlation, though these studies did not consider laterality of site, and thus, for example first and second melanomas on the left and right arms were considered the same site.^9,29,30^ To our knowledge, no previous study has reported the thickness of the subsequent melanoma and time interval between the first and second melanomas by site concordance. In our study, 53% of second primary melanomas were developed on a different body site, highlighting the importance of full body skin examination during the follow-up of patients. We found that the median thickness for subsequent melanomas on the same, contralateral, and different sites were comparable, as well as the median interval between the first and second primary melanomas was comparable between second primary melanomas irrespective of site, except in men, in whom the median interval for melanomas occurring on the same site was shorter.

### Strengths and Limitations

The main strengths of this study include the large population-based sample, the high-quality data, and the use of national registries, ensuring close to complete ascertainment of subsequent melanomas during the time frame of our study. Furthermore, the availability of complete records of patients’ cancer histories dating back to 1953 minimized the misclassification of first vs subsequent melanomas, which can occur in more limited clinical series. Limitations included lack of information on phenotypic characteristics, personal UVR exposure, genetic factors, and the number of follow-up skin examinations.

## Conclusions

The results of this cohort study may have potential implications for a more personalized surveillance of patients with melanoma. Surveillance protocols for patients with melanoma are mainly concerned with identifying disease recurrence, and thus patients are prioritized based on tumor stage at diagnosis.^31^ Current Norwegian guidelines recommend one follow-up after 3 months for a first primary for patients with stage IA disease (most patients) and regular follow-up every 6 months for 5 years for patients with stage IB-IV disease.^32^ However, these criteria do not take into account their material risk of developing a second primary melanoma. Our findings suggest that increased surveillance may be considered for older patients, especially men, for at least the first 3 years after their initial diagnosis, regardless of the characteristics of their first melanoma.

## Notes

Supplement 1. **eTable 1.** Incidence Rates of a Second Primary Melanoma per 1000 Person-Years **eTable 2.** Association Between Age at Diagnosis and Second Primary Melanoma by Sex in Patients Diagnosed With a First Primary Melanoma in Norway Between 2008 and 2020

Supplement 2.
Data Sharing Statement

## References

[1] Urban K, Mehrmal S, Uppal P, Giesey RL, Delost GR. The global burden of skin cancer: a longitudinal analysis from the Global Burden of Disease Study, 1990-2017. JAAD Int. 2021;2:98-108. doi:10.1016/j.jdin.2020.10.013 34409358 PMC8362234

[2] Miller KD, Nogueira L, Devasia T, . Cancer treatment and survivorship statistics, 2022. CA Cancer J Clin. 2022;72(5):409-436. doi:10.3322/caac.21731 35736631

[3] van der Leest RJ, Flohil SC, Arends LR, de Vries E, Nijsten T. Risk of subsequent cutaneous malignancy in patients with prior melanoma: a systematic review and meta-analysis. J Eur Acad Dermatol Venereol. 2015;29(6):1053-1062. doi:10.1111/jdv.12887 25491923

[4] Cust AE, Badcock C, Smith J, . A risk prediction model for the development of subsequent primary melanoma in a population-based cohort. Br J Dermatol. 2020;182(5):1148-1157. doi:10.1111/bjd.18524 31520533 PMC7069770

[5] DiFronzo LA, Wanek LA, Elashoff R, Morton DL. Increased incidence of second primary melanoma in patients with a previous cutaneous melanoma. Ann Surg Oncol. 1999;6(7):705-711. doi:10.1007/s10434-999-0705-0 10560858

[6] Francken AB, Shaw HM, Thompson JF. Detection of second primary cutaneous melanomas. Eur J Surg Oncol. 2008;34(5):587-592. doi:10.1016/j.ejso.2007.06.004 17681449

[7] Goggins WB, Tsao H. A population-based analysis of risk factors for a second primary cutaneous melanoma among melanoma survivors. Cancer. 2003;97(3):639-643. doi:10.1002/cncr.11116 12548605

[8] Nosrati A, Yu WY, McGuire J, . Outcomes and risk factors in patients with multiple primary melanomas. J Invest Dermatol. 2019;139(1):195-201. doi:10.1016/j.jid.2018.07.009 30031745 PMC9191767

[9] Pastor-Tomás N, Martínez-Franco A, Bañuls J, . Risk factors for the development of a second melanoma in patients with cutaneous melanoma. J Eur Acad Dermatol Venereol. 2020;34(10):2295-2302. doi:10.1111/jdv.16341 32163215

[10] Schuurman MS, de Waal AC, Thijs EJM, van Rossum MM, Kiemeney LALM, Aben KKH. Risk factors for second primary melanoma among Dutch patients with melanoma. Br J Dermatol. 2017;176(4):971-978. doi:10.1111/bjd.15024 27596937

[11] Siskind V, Hughes MC, Palmer JM, . Nevi, family history, and fair skin increase the risk of second primary melanoma. J Invest Dermatol. 2011;131(2):461-467. doi:10.1038/jid.2010.298 20944647 PMC3045696

[12] Wiener AA, Schumacher JR, Racz JM, Weber SM, Xu YG, Neuman HB. Incidence of second primary melanoma in cutaneous melanoma survivors. Ann Surg Oncol. 2022;29(9):5925-5932. doi:10.1245/s10434-022-11725-8 35505144 PMC11898002

[13] Ni Y, Watts CG, Scolyer RA, . Risk of developing a second primary melanoma after a first primary melanoma in a population-based Australian cohort. Br J Dermatol. 2023;188(6):814-816. doi:10.1093/bjd/ljad076 36946230

[14] Olsen CM, Pandeya N, Dusingize JC, ; QSkin Study. Risk factors associated with first and second primary melanomas in a high-incidence population. JAMA Dermatol. 2023;159(1):37-46. doi:10.1001/jamadermatol.2022.4975 36416830 PMC9685542

[15] Larsen IK, Småstuen M, Johannesen TB, . Data quality at the Cancer Registry of Norway: an overview of comparability, completeness, validity and timeliness. Eur J Cancer. 2009;45(7):1218-1231. doi:10.1016/j.ejca.2008.10.037 19091545

[16] Statistics Norway. Immigrants and Norwegian-born to immigrant parents. Accessed November 12, 2023. https://www.ssb.no/en/befolkning/innvandrere/statistikk/innvandrere-og-norskfodte-med-innvandrerforeldre

[17] Keung EZ, Gershenwald JE. The eighth edition American Joint Committee on Cancer (AJCC) melanoma staging system: implications for melanoma treatment and care. Expert Rev Anticancer Ther. 2018;18(8):775-784. doi:10.1080/14737140.2018.1489246 29923435 PMC7652033

[18] Bottai M, Discacciati A, Santoni G. Modeling the probability of occurrence of events. Stat Methods Med Res. 2021;30(8):1976-1987. doi:10.1177/09622802211022403 34232832

[19] van der Leest RJ, Liu L, Coebergh JW, . Risk of second primary in situ and invasive melanoma in a Dutch population-based cohort: 1989-2008. Br J Dermatol. 2012;167(6):1321-1330. doi:10.1111/j.1365-2133.2012.11123.x 22759226

[20] Youlden DR, Youl PH, Soyer HP, Aitken JF, Baade PD. Distribution of subsequent primary invasive melanomas following a first primary invasive or in situ melanoma Queensland, Australia, 1982-2010. JAMA Dermatol. 2014;150(5):526-534. doi:10.1001/jamadermatol.2013.9852 25093216

[21] McCaul KA, Fritschi L, Baade P, Coory M. The incidence of second primary invasive melanoma in Queensland, 1982-2003. Cancer Causes Control. 2008;19(5):451-458. doi:10.1007/s10552-007-9106-5 18167620

[22] Ferrone CR, Ben Porat L, Panageas KS, . Clinicopathological features of and risk factors for multiple primary melanomas. JAMA. 2005;294(13):1647-1654. doi:10.1001/jama.294.13.1647 16204664

[23] Levi F, Randimbison L, Te VC, La Vecchia C. High constant incidence rates of second cutaneous melanomas. Int J Cancer. 2005;117(5):877-879. doi:10.1002/ijc.21262 15957164

[24] Moore MM, Geller AC, Warton EM, Schwalbe J, Asgari MM. Multiple primary melanomas among 16,570 patients with melanoma diagnosed at Kaiser Permanente Northern California, 1996 to 2011. J Am Acad Dermatol. 2015;73(4):630-636. doi:10.1016/j.jaad.2015.06.059 26298295

[25] Balamurugan A, Rees JR, Kosary C, Rim SH, Li J, Stewart SL. Subsequent primary cancers among men and women with in situ and invasive melanoma of the skin. J Am Acad Dermatol. 2011;65(5)(suppl 1):S69-S77. doi:10.1016/j.jaad.2011.04.033 22018070

[26] Jones MS, Torisu-Itakura H, Flaherty DC, . Second primary melanoma: risk factors, histopathologic features, survival, and implications for follow-up. Am Surg. 2016;82(10):1009-1013. doi:10.1177/000313481608201034 27779995 PMC5555365

[27] Ghiasvand R, Robsahm TE, Green AC, . Association of phenotypic characteristics and UV radiation exposure with risk of melanoma on different body sites. JAMA Dermatol. 2019;155(1):39-49. doi:10.1001/jamadermatol.2018.3964 30477003 PMC6439571

[28] Robsahm TE, Tretli S. Cutaneous malignant melanoma in Norway: variation by region of residence before and after the age 17. Cancer Causes Control. 2001;12(6):569-576. doi:10.1023/A:1011287918405 11519765

[29] Stam-Posthuma JJ, van Duinen C, Scheffer E, Vink J, Bergman W. Multiple primary melanomas. J Am Acad Dermatol. 2001;44(1):22-27. doi:10.1067/mjd.2001.110878 11148472

[30] Titus-Ernstoff L, Perry AE, Spencer SK, . Multiple primary melanoma: two-year results from a population-based study. Arch Dermatol. 2006;142(4):433-438. doi:10.1001/archderm.142.4.433 16618861

[31] Michielin O, van Akkooi ACJ, Ascierto PA, Dummer R, Keilholz U; ESMO Guidelines Committee. Cutaneous melanoma: ESMO Clinical Practice Guidelines for diagnosis, treatment and follow-up. Ann Oncol. 2019;30(12):1884-1901. doi:10.1093/annonc/mdz411 31566661

[32] Helsedirektoratet. National action program with guidelines for diagnosis, treatment and follow-up of melanoma. Article in Norwegian. Updated July 5, 2023. Accessed December 14, 2023. https://www.helsedirektoratet.no/retningslinjer/melanomer-handlingsprogram

