# Supplementary material for: Incidence and Factors Associated With Second Primary Invasive Melanoma in Norway
Source: JAMA Dermatol. 2024 Feb 28;160(4):402–8. doi: 10.1001/jamadermatol.2023.6251 (PMC10902780; doi:10.1001/jamadermatol.2023.6251)
Supplement: Supplement 1. — eTable 1. Incidence Rates of a Second Primary Melanoma per 1000 Person-Years eTable 2. Association Between Age at Diagnosis and Second Primary Melanoma by Sex in Patients Diagnosed With a First Primary Melanoma in Norway Between 2008 and 2020 [file jamadermatol-e236251-s001.pdf]

Supplemental Online Content

Ghiasvand R, Green AC, Veierød MB, Robsahm TE. Incidence and factors associated with second primary invasive melanoma in Norway. *JAMA Dermatol.* Published online February 28, 2024. doi:10.1001/jamadermatol.2023.6251

eTable 1. Incidence Rates of a Second Primary Melanoma per 1000 Person-Years

eTable 2. Association Between Age at Diagnosis and Second Primary Melanoma by Sex in Patients Diagnosed With a First Primary Melanoma in Norway Between 2008 and 2020

This supplemental material has been provided by the authors to give readers additional information about their work.

© 2024 Ghiasvand R et al. *JAMA Dermatology*.

eTable 1. Incidence rates of a second primary melanoma per 1000 person-years

**Follow up year**	**Incidence rate total**	**Incidence rate women**	**Incidence rate men**
1	16.8 (14.9 – 18.7)	13.5 (11.1 – 15.8)	20.3 (17.4 – 23.3)
2	7.3 (6.0 – 8.6)	4.9 (3.4 – 6.3)	10.0 (7.8 – 12.3)
3	5.4 (4.2 – 6.6)	5.2 (3.6 – 6.9)	5.5 (3.8 – 7.3)
4	5.7 (4.4 – 7.1)	5.7 (3.8 – 7.5)	5.8 (3.8 – 7.8)
5	5.6 (4.2 – 7.1)	4.9 (3.0 – 6.7)	6.6 (4.2 – 8.9)
6	5.5 (3.9 – 7.1)	5.7 (3.4 – 7.9)	5.3 (3.0 – 7.7)
7	5.6 (3.8 – 7.4)	3.3 (1.4 – 5.2)	8.3 (5.1 – 11.6)
8	4.4 (2.6 – 6.2)	3.2 (1.1 – 5.2)	5.9 (2.8 – 9.0)
9	3.7 (1.8 – 5.6)	1.8 (0.4 – 3.6)	6.0 (2.5 – 9.6)
10	4.2 (1.8 – 6.5)	3.1 (0.4 – 5.9)	5.5 (1.4 – 9.6)

© 2024 Ghiasvand R et al. *JAMA Dermatology*.

eTable 2. Association between age at diagnosis and second primary melanoma by sex in patients diagnosed with a first primary melanoma in Norway between 2008 and 2020

	**Subjects (%)**	**Events (%)**	**RR (95% CI)[1]**	**RR (95% CI)[2]**
Age at diagnosis				
Female	9,763 (100)	326 (100)		
< 40 years	1,149 (12)	29 (9)	1.00	1.00
40 – 49 years	1,619 (17)	41 (13)	1.06 (0.66 – 1.71)	1.01 (0.62 – 1.63)
50 – 69 years	1,866 (19)	57 (17)	1.33 (0.85 – 2.08)	1.26 (0.81 – 1.97)
60 – 69 years	2,083 (21)	85 (26)	1.78 (1.16 – 2.71)	1.67 (1.10 – 2.55)
70 – 79 years	1,676 (17)	66 (20)	2.01 (1.30 – 3.12)	1.82 (1.17 – 2.82)
≥ 80 years	1,370 (14)	48 (15)	2.38 (1.50 – 3.79)	2.11 (1.31 – 3.42)
Male	9,433 (100)	440 (100)		
< 40 years	613 (7)	8 (2)	1.00	1.00
40 – 49 years	1,152 (12)	35 (8)	2.41 (1.12 – 5.19)	2.48 (1.15 – 5.36)
50 – 69 years	1,848 (20)	73 (17)	3.38 (1.63 – 7.03)	3.44 (1.66 – 7.16)
60 – 69 years	2,510 (22)	143 (32)	5.05 (2.48 – 10.30)	5.16 (2.52 – 10.58)
70 – 79 years	2,110 (22)	120 (27)	6.01 (2.94 – 12.30)	6.09 (2.60 – 12.55)
≥ 80 years	1,200 (13)	61 (14)	7.12 (3.41 – 14.89)	7.33 (3.46 – 15.51)

[1] Parametric accelerated failure time models with exponential distribution.

[2] Parametric accelerated failure time models with exponential distribution, adjusted for place of residence, tumor characteristics and calendar year of first primary melanoma diagnosis.

© 2024 Ghiasvand R et al. *JAMA Dermatology*.
