# Supplementary material for: Incidence and Factors Associated With Second Primary Invasive Melanoma in Norway
Source: JAMA Dermatol. 2024 Feb 28;160(4):402–8. doi: 10.1001/jamadermatol.2023.6251 (PMC10902780; doi:10.1001/jamadermatol.2023.6251)
Supplement: Supplement 2. — Data Sharing Statement [file jamadermatol-e236251-s002.pdf]

Data Sharing Statement

Ghiasvand. Incidence and Factors Associated With Second Primary Invasive Melanoma in Norway. *JAMA Dermatol*. Published February 28, 2024. doi:10.1001/jamadermatol.2023.6251

Data
Data available: No
